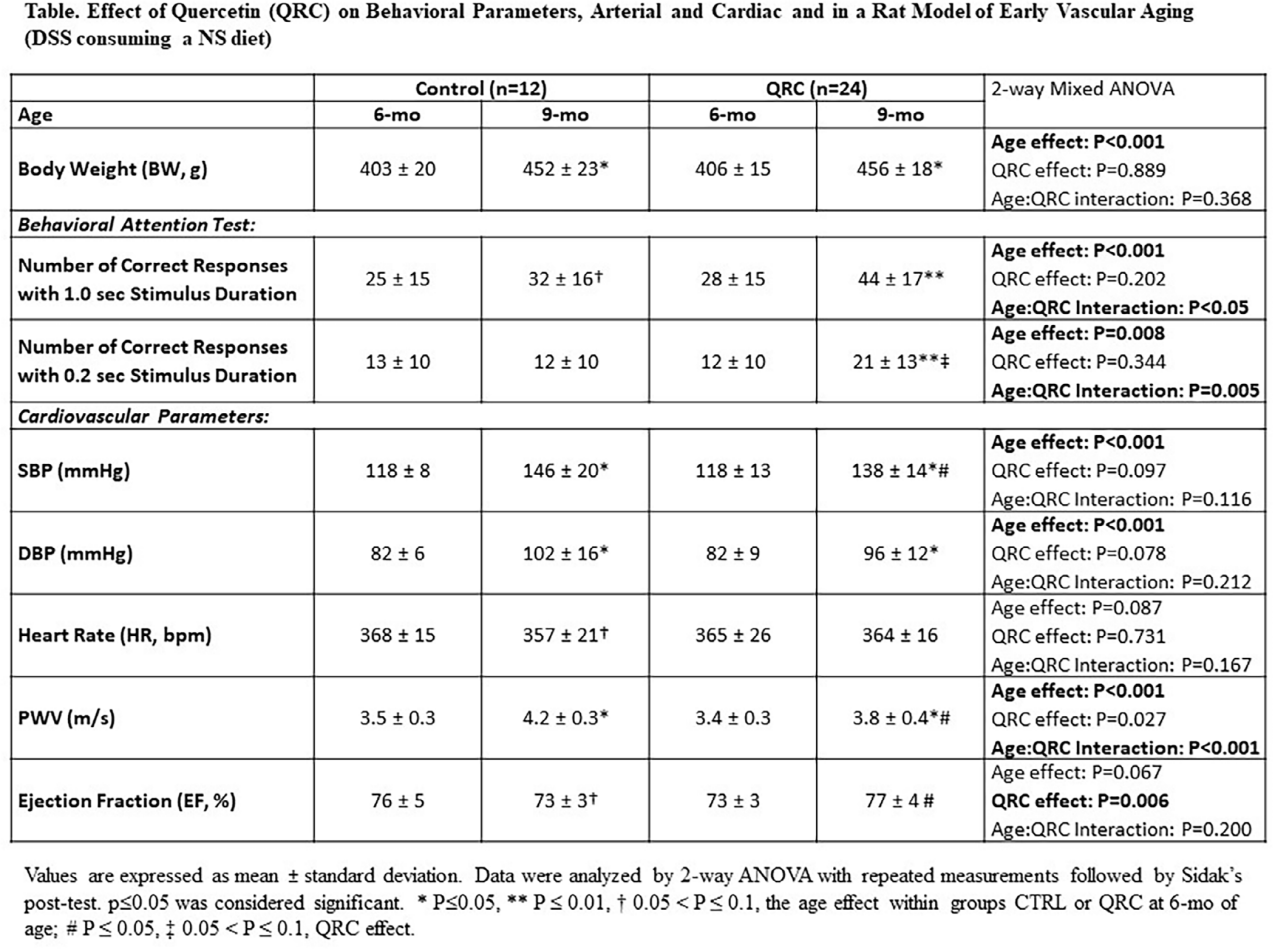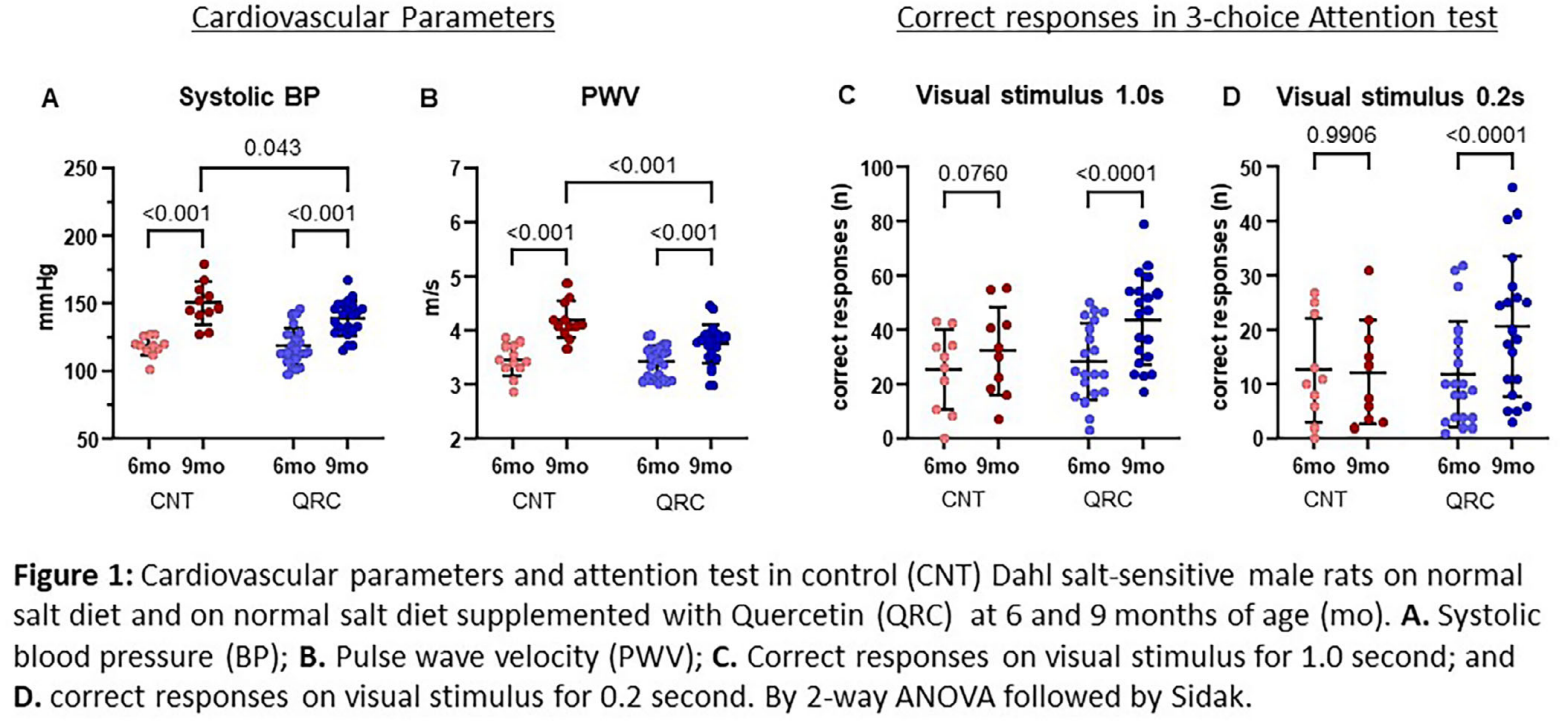# Reduction of central arterial stiffness by senolytic quercetin improves age‐associated decline in cognitive function in a Dahl salt‐sensitive rat model of early vascular aging

**DOI:** 10.1002/alz.087758

**Published:** 2025-01-03

**Authors:** Carla Rocha Dos Santos, Mackenzie J Barnett, Ross A McDevitt, Wen Wei, Valentina I Zernetkina, Ondrej Juhasz, Christopher H Morrell, Edward G Lakatta, Olga V Fedorova

**Affiliations:** ^1^ National Institute on Aging/National Institutes of Health (NIA/NIH), Baltimore, MD USA

## Abstract

**Background:**

Early vascular aging (EVA), manifesting as increases in central arterial stiffness and BP, is associated with cognitive impairment in humans. EVA and cognitive impairment occurs in Dahl salt‐sensitive (DSS) rats consuming a normal salt (NS) diet with an advancing age. Quercetin (QRC), a flavonoid with anti‐oxidant, anti‐inflammatory and senolytic properties, previously shown to reduce salt‐sensitive hypertension in DSS. We hypothesized that QRC will not only reduce EVA but will also improve cognitive function in DSS consuming NS diet.

**Method:**

Six month old male DSS rats were assigned to two groups: a control group (n = 12) on a normal salt diet (0.5% NaCl) and an experimental group (n = 24) receiving the same diet supplemented with QRC (100 mg/kg BW/daily) for a 3‐month period. Systolic and diastolic blood pressure (SBP, DBP), pulse wave velocity (PWV, an index of CAS), echocardiography, and visuospatial attention test were assessed before and after QRC treatment or placebo.

**Result:**

Before treatment, no differences existed between groups. From 6 to 9 months of age in the control group, SBP, DBP, and PWV increased, while heart rate (HR) and ejection fraction (EF) showed a borderline decrease, and attention behavior showed a borderline increase for 1.0s of stimulus duration and no change for shorter visual stimulus, 0.2s (Figure and Table). QRC treatment reduced SBP, PWV, and increased EF and attention. The QRC effect was to increase the number of correct responses for 0.2s of stimulus and was negatively correlated with PWV (Pearson r = ‐0.420, P = 0.02) but not with BP, indicating that CAS is linked to cognitive function.

**Conclusion:**

QRC supplementation demonstrated the ability to reduce EVA and enhance cognition in DSS rats, as indicated by attention behavior. Despite BP and CAS reflecting EVA, the association between attention decline and PWV, but not BP, implies that CAS is linked to cognitive function through mechanisms not directly related to BP. Further exploration is required to understand the mechanistic basis of QRC effects on EVA in DSS rats.

Supported by NIA/NIH/IRP